# Catalepsy

**Published:** 1853-04

**Authors:** W. P. Jones


*Catalepsy.* By Dr. W. P. Jones.—Several years ago, while in one of our Western States, I accepted a courteous invitation from Dr. W., to visit with him an interesting young lady, whom he represented to be in a cataleptic condition.

It was probably 11 o'clock, A. M., when we entered the room of the patient, who was in bed—her eyes, and seemingly her mind, steadily fixed upon some object on the ceiling—her pulse exceedingly slow, respiration perhaps twelve to the minute. This being her condition, of course our entrance to her room, and nearer approach to her bed, had been unobserved by other than the family and friends; and here, in company with Dr. A. (consulting physician), we carefully observed her then condition.

During the intervals of respiration, Dr. W. (who, by the way, was not a believer in animal magnetism) placed his hand over hers, and by slowly raising his hers followed; this he did frequently, and under similar circumstances, with like results. I exerted more control over her than did Dr. W.; Dr, A., however, exerted greatly more influence than either of us. By placing his hand at the distance of an inch from her forehead, he could bring her almost to the sitting posture; she, however, fell like lead the moment she commenced breathing. Without touching at all, but by merely bringing my index finger in close proximity to either of hers, they were flexed and extended at will. And by placing her hands in any position which caprice or fancy might suggest, there they would remain until the next inspiration.

This condition was uniformly succeeded by the most frightful convulsions, which lasted, usually, from ten to fifteen minutes; after which she gradually resumed a state of tranquility and consciousness. Notwithstanding the treatment which the most skilful physicians were competent to adopt, these paroxysms were of almost daily recurrence for several months, when she slowly though entirely recovered.—*Nashville Southern Journal.*

